# Poly(quercetin)-bismuth nanowires as a new modifier for simultaneous voltammetric determination of dihydroxybenzene isomers and nitrite

**DOI:** 10.1039/c7ra11132k

**Published:** 2018-01-03

**Authors:** M. Tohidinia, M. Farsadrooh, S. Bahmanzadeh, N. Sabbaghi, M. Noroozifar

**Affiliations:** Department of Chemistry, University of Sistan and Baluchestan P.O.Box 98135-674 Zahedan Iran mnoroozifar@chem.usb.ac.ir

## Abstract

Dihydroxybenzene isomers and nitrite, NO_2_^−^, are present in the environment as highly toxic compounds and cause human cancer. In this study, for the first time poly(quercetin) (PQ) was synthesized from the reaction between quercetin (Q) and hydroquinone (HQ) as a linker. Bismuth nanowires (BNWs) were synthesized using a solvothermal technique and then the BNWs and PQ were used for preparation of a novel modified graphite paste electrode (GPE/PQ–BNWs) for simultaneous determination of dihydroxybenzene isomers; HQ, catechol (CC), resorcinol (RS) in the presence of NO_2_^−^. The product was characterized using X-ray diffraction, field emission scanning electron microscopy and Fourier transform infrared spectroscopy. The electrochemical response characteristics of the modified GPE toward mix HQ, CC, RS and NO_2_^−^ were investigated by cyclic voltammetry, differential pulse voltammetry and electrochemical impedance spectroscopy. Under the optimum conditions, detection limits of 0.12, 0.2, 0.82 and 4.5 μM were obtained for HQ, CC, RS and NO_2_^−^, respectively. Moreover, the GPE/PQ–BNWs were applied to determine HQ, CC, RS and NO_2_^−^ in water samples with satisfactory results.

## Abstract 2

Dihydroxybenzene isomers and nitrite, NO_2_^−^, are present in environment as the high toxic compounds and cause human cancer. A novel GPE/PQ–BNWs for simultaneous determination dihydroxybenzene isomers; HQ, CC, RS in presence of NO_2_^−^.

## Graphical abstract

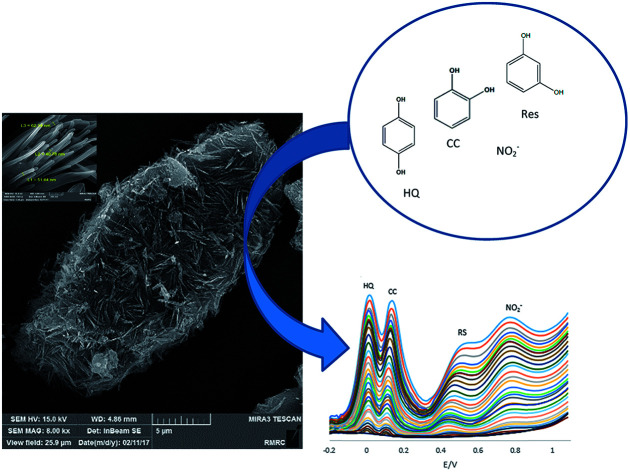

## 1. Introduction

The dihydroxybenzene isomers, hydroquinone (HQ), catechol (CC) and resorcinol (RS), exist in the environment as high toxicity and low degradability chemicals of polluted water.^1,2^ They are used in a large number of industries, such as tanning, cosmetics, dyes, coal mining, oil refinery, paint, polymer and pharmaceutical preparation.^2,3^ Their unauthorized concentration in the environment can show damaging effects on human health, animals, plants and aquatic life.^4^ They can enter the body *via* the skin, respiratory and digestive apparatus to erode skin and mucosa and even inhibit the nervous system.^1^ High concentrations of HQ could cause headaches, exhaustion, tachycardia and kidney damage. The high concentration of CC could induce DNA harm and cause human cancer.^1,5^ Inhalation of high concentrations of RS can lead to death.^6^ They have been included in the list of priority pollutants by international bodies such as the US Environmental Protection Agency (EPA).^7^ Nitrite is an important environmental pollutant in fresh, brackish, and marine waters due to a range of factors such as aquaculture wastes, urban sewage, and agricultural runoff.^8^ It is a main cause for formation of highly carcinogenic *N*-nitrosamines compound, which are known to be high cancers in human bodies.^9–11^ Both the phenolic compounds and nitrite as environmental pollutants might coexist in one sample, so, it is really required to monitor or quantitate them simultaneously in a complex system.^12^ Various techniques such as spectrophotometry,^13^ fluorescence,^14^ chemiluminiscence,^15^ chromatography^16,17^ and electrochemistry^6,7,18^ have been used for determination of HQ, CC, RS and NO_2_^−^. The advantages of electrochemistry are quick response, inexpensive instrumentation, simple operation, time saving, high sensitivity and great selectivity.^19,20^ Recently, just Yang *et al.*^12^ and Wang *et al.*^21^ have used gold nanoparticle–graphene nanohybrid bridged 3-amino-5-mercapto-1,2,4-triazole-functionalized multiwall carbon nanotubes and gold nanoparticles loaded on poly-3-amino-5-mercapto-1,2,4-triazole-multiwall carbon nanotubes film modified electrode for simultaneous determination of HQ, CC, RS and NO_2_^−^.

Quercetin (Q) is a flavanid which can found in many fruits, vegetables, leaves, and grains. It can be used as a part in supplements, beverages, or food.^22^ In 2013, Sahiner reported synthesis polyquercetin (PQ) from reaction between L–α lecithin as surfactant and cyclohexane as organic phase.^23^ In other hand, there has been considerable interest in fabricating nanostructured materials such as multilayers, nanowires, and nanoparticles, also other composite materials. Arrays of nanowires such as bismuth nanowires (BNWs) are a recent type of nanostructures that exhibit quasi-1D characteristics. Several methods have been reported for the preparation of BNWs.^24–27^

In this study, for the first time PQ was synthesized from reaction between Q and HQ and then used for preparation of a novel modified graphite paste electrode with PQ and BNWs for simultaneous determination of HQ, CC, RS and NO_2_^−^. To the best of our knowledge, no study has been reported on the electroanalysis and simultaneous determination of HQ, CC, RS and NO_2_^−^ using a modified graphite paste with PQ/BNWs. The consequences demonstrated a wide concentration ranges, low detection limits and excellent selectivity. In addition, the proposed method successfully was applied for the simultaneous determination of HQ, CC, RS and NO_2_^−^ in water samples.

## 2. Experimental

### 2.1. Reagents

CC, RS, HQ and NO_2_^−^ were bought from Sigma-Aldrich and sodium nitrite, sodium hydroxide, hydrogen chloride, phosphoric acid, potassium dihydrogen phosphite, potassium hydrogen phosphite, bismuth nitrate, ethylene diamine, acetone, alcohol, graphite powder and paraffin oil were purchased from Merck. All reagents with analytical grade were used as received without any purification.

### 2.2. Apparatus

Cyclic voltammetry (CV) and differential pulse voltammetry (DPV) were performed on a potentiostat/galvanostat (SAMA 500, electroanalyzer system, Iran) with a three-electrode system that the modified electrode as the working electrode, a platinum electrode as the counter electrode and an Ag/AgCl/saturated KCl as the reference electrode. Electrochemical impedance spectroscopy (ESI) was performed with an Autolab PGSTAT 128N (EcoChemie, Netherlands) potentiostat/galvanostat controlled by NOVA 1.11 software. Field Emission Scanning Electron Microscopy (FESEM) and Energy Dispersive X-ray Analysis (EDS) were carried out using MIRA3 TESCAN and SAMX electron microscope, respectively. X-ray diffraction data were carried out using a Phillips XPERT diffractometer. Fourier transform-infrared spectroscopy (FT-IR) spectra were measured in transmission mode using Valor III (JASCO) equipped with a MCT detector. A Metrohm pH meter, model 744 was also used for pH measurements.

### 2.3. Preparation of experimental solutions

A series of buffer solutions with H_3_PO_4_, KH_2_PO_4_ and K_2_HPO_4_ were prepared and pHs were adjusted using NaOH (0.1 M) and HCl (0.1 M) in the range from 2.0 to 9.0. The solution of Q solution (0.01 M) was prepared by dissolving the solid in a small volume of 0.1 M NaOH solution and diluted to reach favorite concentration. The stock solutions of 0.01 M or 0.1 M HQ, 0.01 M CC, 0.1 M RS and 0.1 M NO_2_^−^ were freshly prepared by dissolving HQ, CC, RS and NO_2_^−^ in double stilled water (DDW). All electrolyte solutions were prepared with DDW and deoxygenated with nitrogen bubbling before each voltammetric experiment at room temperature (RT).

### 2.4. Preparation of BNWs

The solvothermal technique was used for Bi nanowires preparation.^27^ In this technique, 2 g of Bi(NO)_3_·5H_2_O and 5 ml acetone were added into 40 ml pure ethylene diamine and mixed at RT. The insoluble precipitate of above mixture was stirred strongly for 10 min and then transferred into a stainless steel autoclave with an 80 ml internal Teflon vessel. The autoclave was sealed and maintained at 160 °C for 6 h, then cool to RT. The black product was washed three times with alcohol and DDW, respectively. Finally, the product was dried in air at 50 °C for 30 min.

### 2.5. Preparation of PQ

**Scheme 1:**
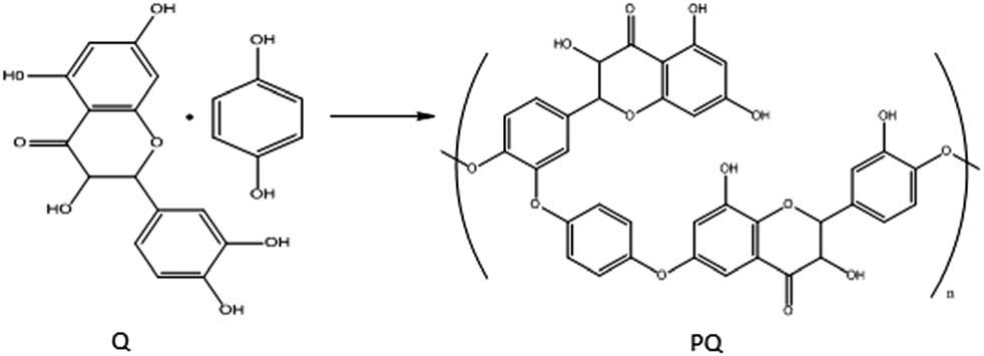
Synthesis of PQ from Q and HQ.

The PQ was prepared by adding 2.5 ml solution of 0.1 M HQ to 2.5 ml solution 0.01 M Q, and the resulting solution was stirred for 3 hours at RT. Solution color was turned from orange to black, indicating that Q crosslinking with HQ and PQ is produced. Then PQ solution was dried at RT. The possible mechanism for synthesis of PQ was shown in Scheme 1.

### 2.6. Preparation of modified graphite paste electrode with PQ–BNWs

The PQ–BNWs was prepared by adding 2.5 ml solution of 0.1 M HQ to 2.5 ml solution 0.01 M Q and BNWs (1 mg ml^−1^) then the resulting solution was stirred for 3 hours at RT. Then PQ–BNWs solution was dried at RT. The GPE/PQ–BNWs was prepared by mixing 0.006 g PQ/BNWs and 0.194 g of graphite powder and 0.4 ml paraffin, and using a mortar and pestle. The mixture was ground until a uniform paste was obtained. The resulting paste was then put in the bottom of a glass pipe with an inner diameter of 4 mm and a length of 10 cm. Electrical connectivity was made by a copper wire lead fitted into the glassy pipe. A new surface was obtained by pushing some of the paste out of the pipe and polishing the end with a weighing paper. Moreover, the GPE/PQ prepared in the same way without adding BNWs to the mixture and unmodified GPE was prepared in the same way without adding PQ and BNWs to the mixture. These GPEs were used for the aim of comparison.

## 3.1. Morphological, XRD and FTIR characterization

**Fig. 1:**
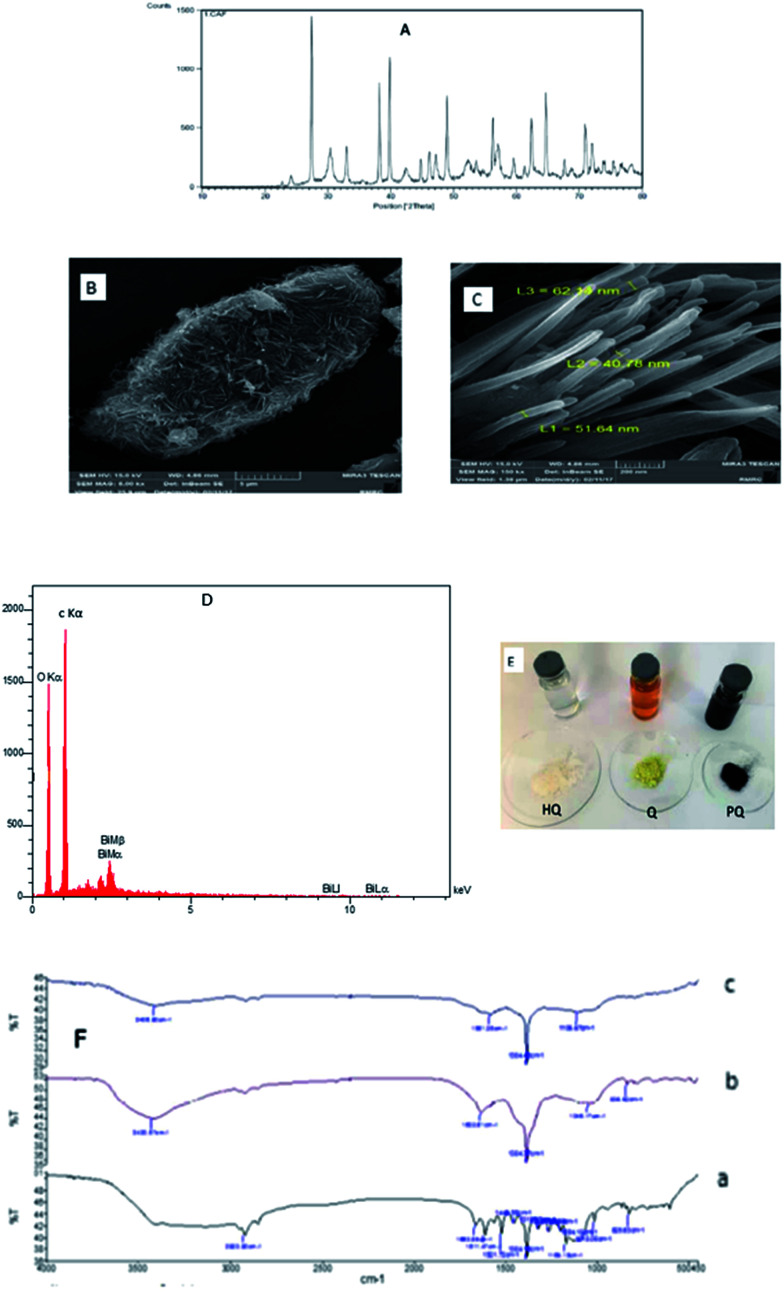
(A) XRD images of bismuth nanowires, (B) FESEM images of PQ–BNWs and (C) BNWs, (D) EDX of PQ–BNWs, (E) photograph of solution and solid of HQ, Q and PQ and (F) FT-IR of (a) the GPE, (b) the GPE/PQ and (c) GPE/PQ–BNWs.

Fig. 1A shows XRD pattern of the product. Compared the d values (*d* = 3.93, 3.70, 3.26, 2.35, 2.26, 2.02, 1.96, 1.86, 1.64, 1.55, 1.51, 1.49, 1.44, 1.38, 1.33, 1.31, 1.28, 1.26, 1.18, 1.14, 1.12, 1.09, 1.07, 1.05, 1.02, 0.98, 0.97, 0.96, 0.88, 0.86 Å) with those in standard JCPDS cards for reference pattern for single-crystalline bismuth nanowires (JCPDS, 01-0669), the peaks can be well indexed with the reflections (*h k l*) of (0 0 3), (1 0 1), (0 1 2), (1 0 4), (1 1 0), (0 1 5), (1 1 3), (2 0 2), (0 2 4), (1 0 7), (2 0 5), (1 1 6), (1 2 2) (0 1 8), (2 1 4), (3 0 0), (0 2 7), (1 2 5), (2 0 8), (1 1 9), (2 1 7), (2 2 3), (3 1 2), (1 2 8), (1 3 4), (4 0 1) (3 1 8), (2 2 9) of Bi rhombohedral phase [space group: *R*3̄*m*(166). Lattice constants calculated from the diffraction data are *a* = *b* = 4.536 Å and *c* = 11.850 Å, which are compatible with the literature values (*a* = *b* = 4.546 Å, *c* = 11.852 Å). This XRD pattern indicates that the reduction of Bi^3+^ to elemental Bi is complete and pure metal Bi products were obtained. Fig. 1B was shown FESEM of the PQ–BNWs and a typical FESEM image of BNWs was presented in Fig. 1C. The product consists of nanotubes with uniform diameter of 40–60 nm and lengths up to 400 nm. It is interesting that all the tubes are straight and most of them are aligned together to form nanotube bundles. Also, the EDX spectrum image of PQ–BNWs was shown at Fig. 1D that shows peak Bi in the product. A photograph of solution of HQ, Q and PQ and solid HQ, Q and product PQ were shown in Fig. 1E. Based on these photographs, the color of Q was changed from yellow to black color after reaction with HQ. The FT-IR of Q, PQ and PQ–BNWs were shown in Fig. 1F. Based on this figure, the spectrum of PQ is simpler than spectrum of Q, which can be symmetric matter.^28^ Decreasing the number of wave carbonyl group of 1632.61 cm^−1^ in PQ spectrum to 1581.38 cm^−1^ in PQ–BNWs spectrum could indicate is due to the interaction of BNWs with oxygen in carbonyl group.

## 3.2. Electrochemical impedance spectroscopy study

**Fig. 2:**
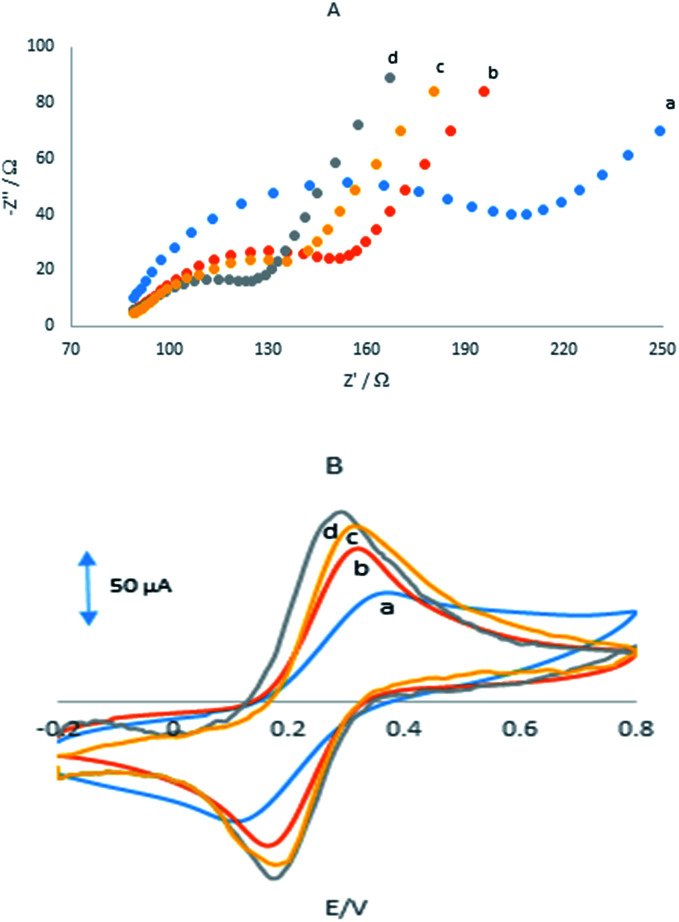
(A) Nyquist plots showing the step-wise modification of (a) GPE, (b) GPE/PQ, (c) GPE/BNWs and (d) GPE/PQ–BNWs. Electrochemical measurements were performed in 10 mM Fe(CN)_6_^3−/4−^ prepared in 0.1 M KCl. EIS was analyzed over a frequency range of 0.1 Hz to 10 kHz, (B) CVs of (a) the GPE, (b) the GPE/PQ, (c) GPE/BNWs and (d) GPE/PQ–BNWs in 1 mM Fe(CN)_6_^3−/4−^ prepared in 0.1 M KCl at scan rate 100 mV s^−1^.

Electrochemical impedance spectroscopy was employed to measuring the impedance value of the electrode surface during the process of frequency variation that is able to offer various properties of the interface of the electrode and solution. By using Fe(CN)_6_^3−/4−^ redox couple as the electrochemical probe, the Nyquist plots of different electrodes were shown in Fig. 2A with the frequencies range from 0.01 Hz to 100 kHz.

It can be seen that at the GPE, GPE/PQ, GPE/BNWs and GPE/PQ–BNWs a semicircle with *R*_ct_ about 125, 66, 51 and 31 Ω was obtained, respectively. These results indicate that a enhancing the rate of electron transfer for Fe(CN)_6_^3−/4−^ redox reaction occurred due to the presence of nanowires. As we expected the charge transfer resistance was changed to low value in the presence of PQ–BNWs in GPE/PQ–BNWs electrode. Fig. 2B displayed the cyclic voltammograms of for the GPE, GPE/PQ and GPE/PQ–BNWs electrodes in the above-mentioned solution which was in good agreement with the results obtained from of the EIS spectrum. Based on this figure, the current intensity of the electrodes changed as GPE/PQ–BNWs > GPE/BNWs > GPE/PQ > GPE. These results showed that the BNWs decreased the electron transfer resistance of GPE/PQ–BNWs in comparison of GPE/PQ as well as GPE electrodes surface.

## 3.3. Electrochemical behaviour of the electrodes

**Fig. 3:**
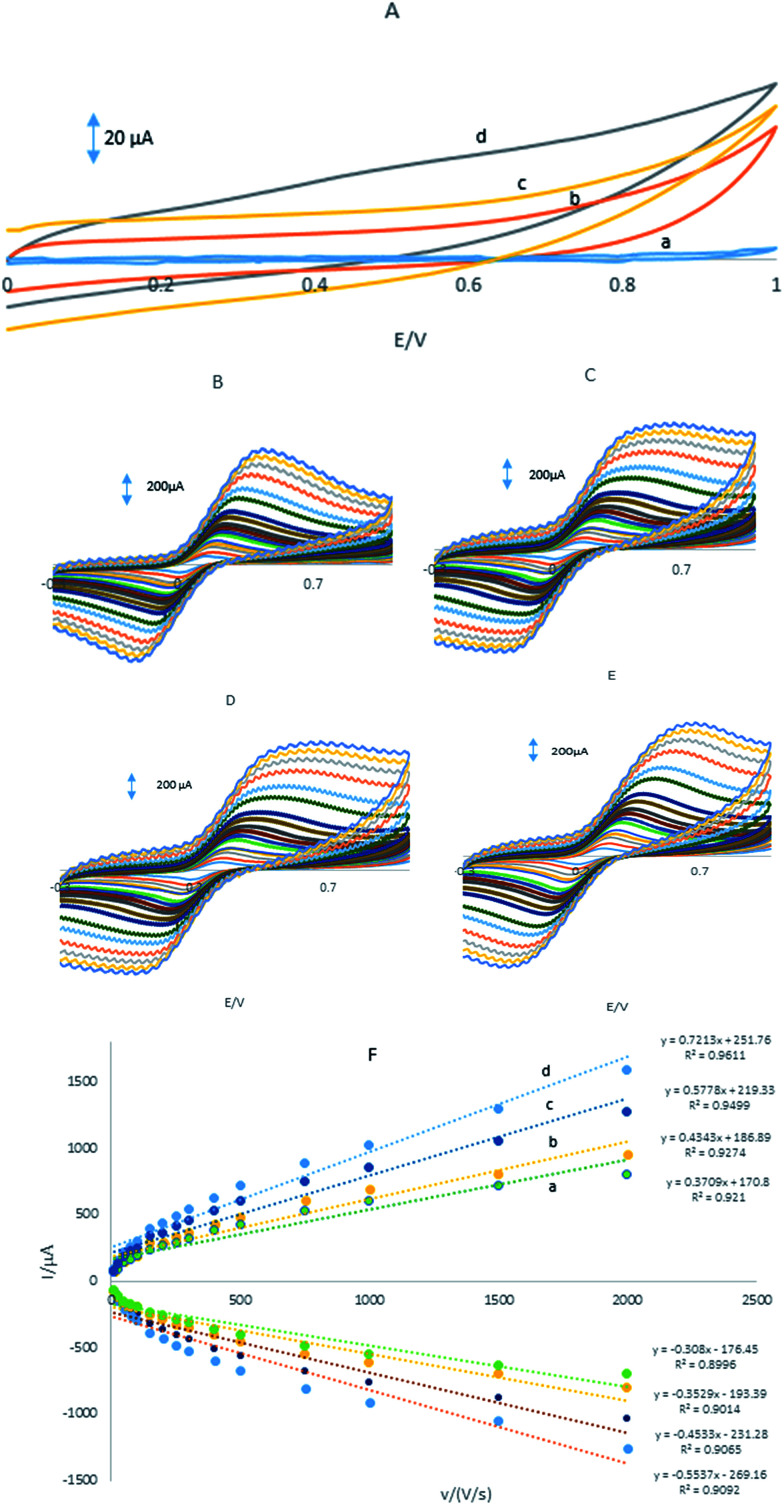
(A) CVs in pH 8.0 PBS (a) GPE, (b) GPE/PQ, (c) GPE/BNWs and (d) GPE/PQ–BNWs, CVs of (B) the GPE, (C) the GPE/PQ, (D) GPE/BNWs and (E) GPE/PQ–BNWs in 1 mM Fe(CN)_6_^3−/4−^ prepared in 0.1 M KCl (F) the plot of peak current *vs.* the square root of scan rate for (a) GPE, (b) GPE/PQ, (c) GPE/BNWs and (d) GPE/PQ–BNWs.

Fig. 3A showed CVs were obtained in the PBS at the GPE/PQ–BNWs, the GPE/PQ and unmodified GPE. At the GPE/PQ–BNWs the field current is greater than the GPE/PQ and unmodified GPE. Based on this figure the charge transfer of the GPE/PQ–BNWs is more. Electrochemical measurements of the GPE/PQ–BNWs, GPE/BNWs, GPE/PQ and GPE were analyzed for the anodic peak current (*I*_pa_) of the respective cyclic voltammograms obtained in the presence of 1.0 mM of Fe(CN)_6_ in KCl 0.1 M of supporting electrolyte (Fig. 3B–D). At Fig. 3E all assays were performed by cyclic voltammetry between potentials of −0.3 to 1 V as a probe at different scan rates. For a reversible process, the Randles–Sevcik equation^29^ can be used.

1 *I*_pa_ = 2.69 × 10^5^*n*^3/2^*AC*_0_*D*_R_^1/2^*v*^1/2^

where *I*_pa_ refers to the anodic peak current, *n* is the electron transfer number, *A* is the surface area of the electrode, *D*_R_ is the diffusion coefficient, *C*_0_ is the concentration of K_3_Fe(CN)_6_ and *v* is the scan rate. For 1 mM K_3_Fe(CN)_6_ in the 0.1 M KCl electrolyte, *n* = 1 and *D*_R_ = 7.6 × 10^−6^ cm s^−1^; the microscopic areas were calculated from the slope of the *I*_pa_–*v*^1/2^ relation. For unmodified GPE, GPE/PQ and GPE/PQ–BNWs, the electrode surface was found to be 0.0233, 0.0226 and 0.0386 cm^2^, respectively. Therefore, the results indicated the presence of BNWs together, greatly improved the effective area of the electrode and cooperate to increase the conductivity of the sensor.

## 3.4. Electrochemical behavior of HQ, CC, RC and NO_2_^−^ at different electrodes

**Fig. 4:**
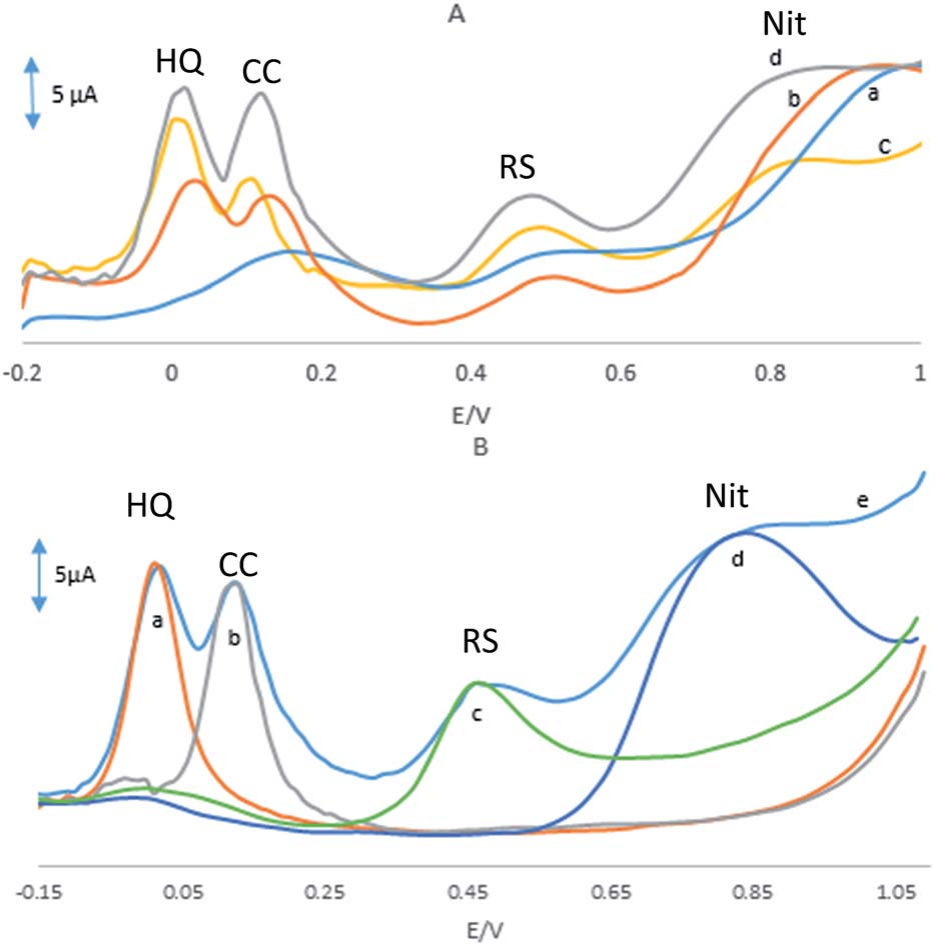
(A) DPVs in pH 8.0 PBS in presence of a mixed solution of 20 μM HQ, 30 μM CC, 50 μM RS and 0.1 mM NO_2_^−^ at (a) GPE, (b) GPE/PQ, (c) GPE/BNWs and (d) GPE/PQ–BNWs. (B) DPVs of the GPE/PQ–BNWs with scan rate of 50 mV s^−1^ in pH 8.0 PBS and in the presence of (a) 20 μM HQ, (b) 30 μM CC, (c) 50 μM RS and (d) 0.1 mM NO_2_^−^ and (e) a mixed solution of (a–d).

Fig. 4A shows the DPVs of a mixture of 20 μM HQ, 30 μM CC, 50 μM RS and 0.1 mM NO_2_^−^ at the surface of the GPE, GPE/PQ, GPE/BNWs and GPE/PQ–BNW in a 0.1 M phosphate buffer solution of pH 8.0 at scan rate of 50 mV s^−1^. As shown in Fig. 4A(a), there is a broad and merge oxidation peak at potential 0.017 V for HQ and CC, a weak peak for RS at potential 0.5 V and there is a peak at potential 0.97 V for NO_2_^−^ at the GPE. As shown in Fig. 4A(b), there are oxidation peaks at 0.04, 0.14, 0.5 and 0.94 V at the GPE/PQ for HQ, CC, RS and NO_2_^−^ respectively. Based on these results, the oxidation peaks of HQ, CC and RS on the surface of GPE and GPE/PQ are too weak to detect and no acceptable different currents are visible. Based on Fig. 4A(c), there are four oxidation peaks at 0.02, 0.12, 0.51 and 0.93 V at the GPE/BNWs and also four oxidation peaks at 0.01, 0.11, 0.45 and 0.80 V at the GPE/PQ–BNWs (see Fig. 4A(d)) for HQ, CC, RS and NO_2_^−^, respectively. Based on Fig. 4A(c and d), the oxidation peak currents of HQ, CC, RS and NO_2_^−^ were increased with a negative peak potential shift for the GPE/PQ–BNWs in compare with GPE/BNWs. So, these results were indicated that the GPE/PQ–BNWs improved the separation of oxidation peak currents and caused a considerable enhancement in the kinetic of anodic peak current of HQ, CC, RS and NO_2_^−^ in compare with the GPE, GPE/PQ and GPE/BNWs. All improvement in the analyte signals can be explained with synergic effect of PQ–BNWs.

Also, the selective determinations of HQ, CC, RS and NO_2_^−^ on the GPE/PQ–BNWs electrode were studied by the comparison DPVs of the simultaneous and individual determinations of these four compounds. The results are displayed in Fig. 4B, that indicating the oxidation processes of these four compounds are independent and therefore, simultaneous determination of HQ, CC, RS and NO_2_^−^ on the GPE/PQ–BNWs is possible without any significant interference. The resulting selectivity and sensitivity of the four anodic peak currents is sufficient to achieve the accurate simultaneous determination of HQ, CC, RS and NO_2_^−^ in mixture samples.

## 3.5. Influence of pH on the simultaneous oxidation of HQ, CC, RS and NO_2_^−^

**Fig. 5:**
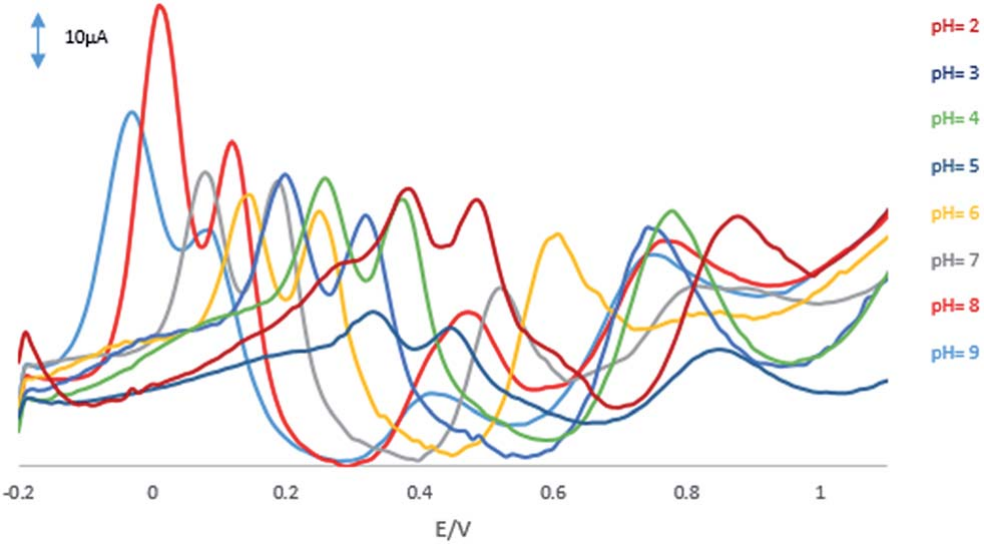
DPVs of the GPE/PQ–BNWs of 55 μM HQ, 55 μM CC, 110 μM RS and 1 mM NO_2_^−^ at scan rate of 50 mV s^−1^ with pH values 2 to 9.

Fig. 5 shows the effect of pH on the GPE/PQ–BNWs of 55 μM HQ, 55 μM CC, 110 μM RS and 1 mM NO_2_^−^ in 0.1 M PBS at the range of 2.0–9.0.

Based on this figure, with increasing the pH, the peak currents and separation peaks of HQ, CC, RS and NO_2_^−^ increased, and the potentials shifted negatively due to the participation of protons in the electrode reaction. The GPE/PQ–BNWs achieved the optimal response to HQ, CC, RS and NO_2_^−^ at pH 8.0. The potentials of HQ, CC, RS and NO_2_^−^ followed the linear regression equations with:

2 *E*_p(HQ)_ = −0.059pH + 0.493, *R*^2^ = 0.9871

3 *E*_p(CC)_ = −0.057pH + 0.591, *R*^2^ = 0.9913

4 *E*_p(RS)_ = −0.059pH + 0.941, *R*^2^ = 0.9783

5 *E*_p(NO_2_^−^)_ = −0.027pH + 0.995, *R*^2^ = 0.9918

According to the formula, d*E*_p_/dpH = −2.303(*mRT*/*nF*), where *m* and *n* are the number of proton and electron, respectively.^30^ The slope of the equations of HQ, CC and RS are close to the theoretical value of 0.059 V pH^−1^.^31^ It can be concluded that equal number of electrons and protons (*m* = *n* = 1, 2, 3, …) are involved in the electrode reactions. This conclusion is in accordance with the known two electrons and two protons electrochemical reactions of HQ and CC at the surface of GPE/PQ–BNWs, which are illustrated in eqn (6) and (7).6
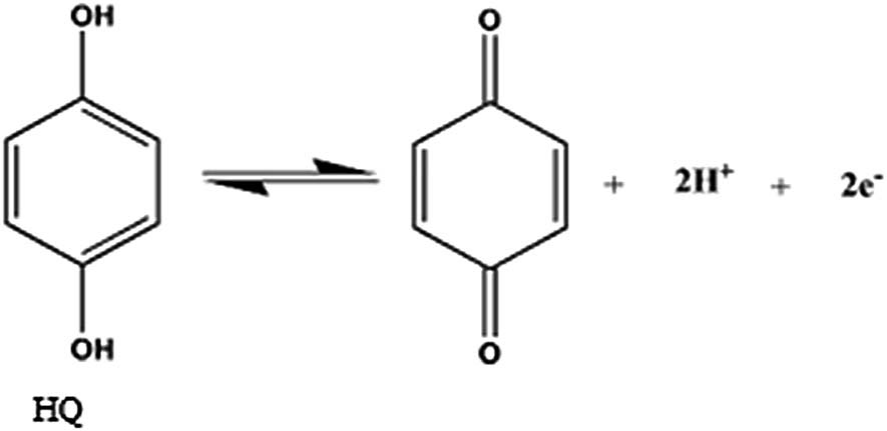
7
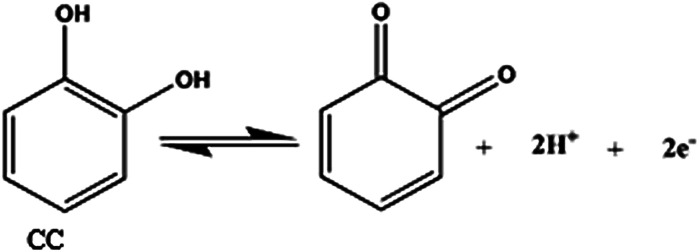


Unlike HQ and CC, in the RS molecule, between π electrons and the hydroxyl group, they are not resonant because they are inside the benzene ring. It means that more oxidation potential (over potential) is required (see Fig. 4), and the oxidation reaction for RS is different from that of HQ and CC and it is one kind of polymerization of RS groups (keto–enol tautomerrism reaction). This reaction for RS is shown in eqn (8). Based on this reaction, after a one electron and one proton electrochemical reaction, three kinds radicals formed and then they react with another RS to form dimers and these dimers are more easily oxidized than the monomer.^32^ The dimers that will predominate are those which are ether linked and those that are carbon linked. The peroxide linked dimers will be highly unstable and will break up as soon as they are formed.^33^ The bond dissociation energies of oxygen–oxygen bonds are less than half that of carbon–carbon bonds. For phenol the predominant form of bonding in the polymer would be carbon–carbon, but for more highly substituted phenols a greater proportion of the bonds tend to be of the ether variety.^34^ The same results reported by Gattrel and Krik,^35^ too.8
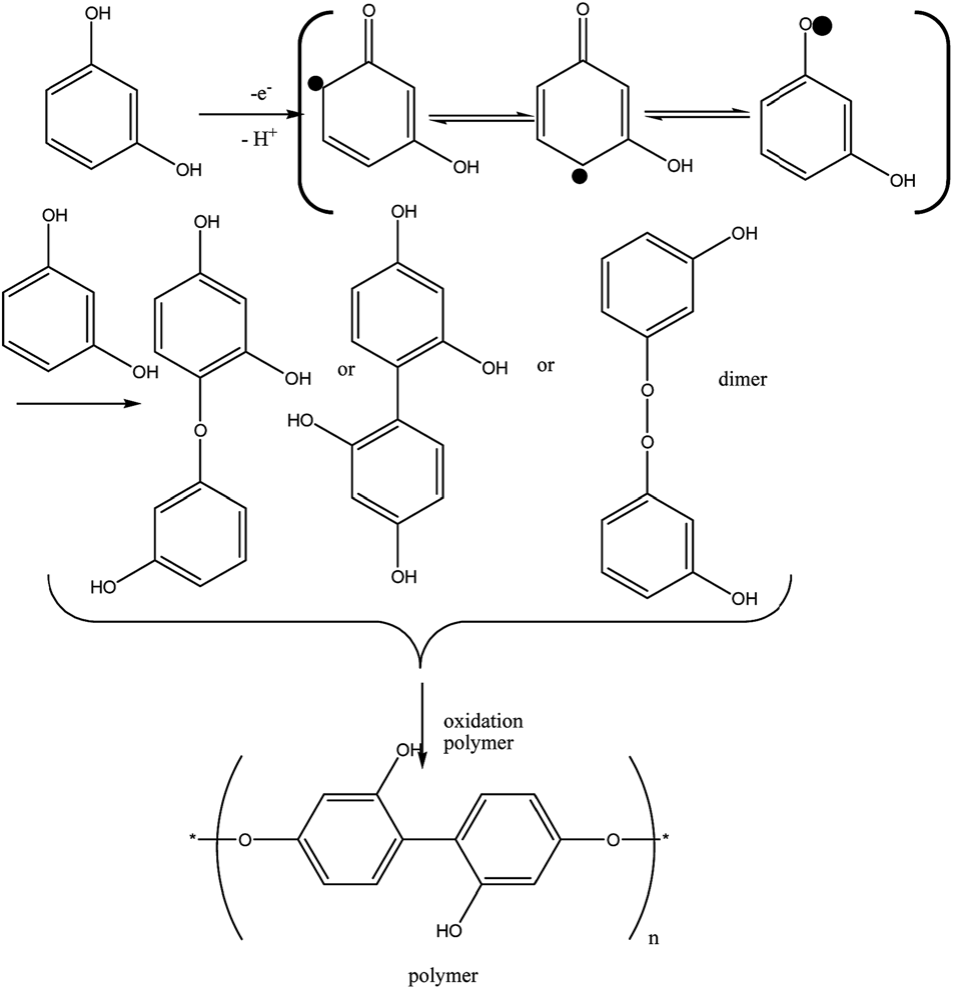


The slope of NO_2_^−^ is near to theoretical slope 0.028 V pH^−1^, indicating that the electrochemical redox of NO_2_^−^ involves an unequal number of protons and electrons should be a two electrons and one proton process which are illustrated in eqn (9).

9 NO_2_^−^ + H_2_O–H^+^ → HNO_3_

## 3.6. Linear range, detection limit and simultaneous determination

**Fig. 6:**
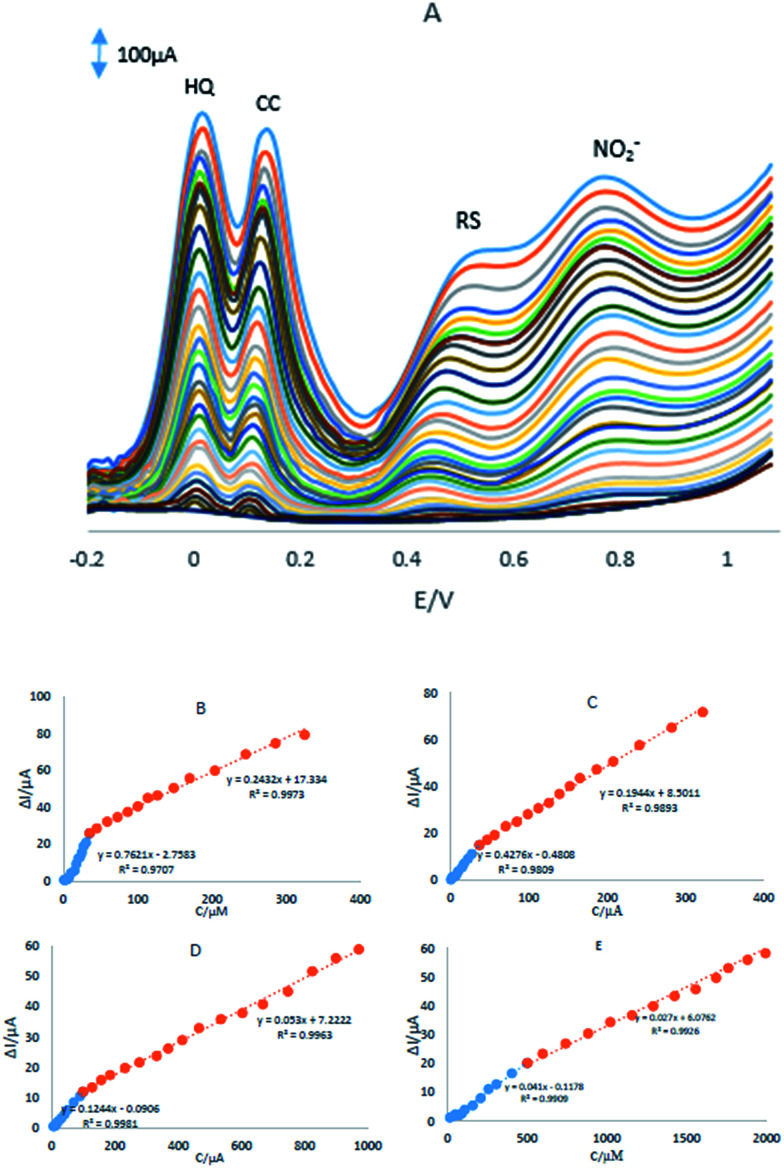
(A) DPVs of the GPE/PQ–BNWs in pH 8.0 PBS containing different concentrations of HQ, CC, RS and NO_2_^−^. Plots of *I*_p_*vs.* concentration (B) HQ, (C) CC, (D) RS and (E) NO_2_^−^.

DPV was used for simultaneous determination of HQ, CC, RS and NO_2_^−^ and the results were shown in Fig. 6A. In order to obtain the best sensitivity under the specific conditions, an amplitude scan rate of 50 mV and pH 8.0 were selected. The responses were linear with HQ concentration consisted of two linear segments with slopes of 0.7621 and 0.2432 μA μM^−1^ in the concentration ranges from 0.76 μM to 35.1 μM and 35.1 μM to 324.7 μM, respectively (Fig. 6B).

The responses were linear with CC concentration consisted of two linear segments with slopes of 0.4276 and 0.1944 μA μM^−1^ in the concentration ranges from 0.74 μM to 37.0 μM and 37.0 μM to 322.0 μM, respectively (Fig. 6C). Linear with RS concentration consisted of two linear segments with slopes of 0.1244 and 0.0530 μA μM^−1^ in the concentration ranges from 4.99 μM to 98.3 μM and 98.3 μM to 970.7 μM, respectively (Fig. 6D) and the responses were linear with NO_2_^−^ concentration consisted of two linear segments with slopes of 0.041 and 0.027 μA μM^−1^ in the concentration ranges from 14.9 μM to 501.2 μM and 501.2 μM to 2392.6 μM, respectively (Fig. 6E). The detection limits were determined to be 0.12 μM, 0.2 μM, 0.82 μM and 4.5 μM for HQ, CC, RS and NO_2_^−^, respectively.

Based on the Fig. 6B–E, the difference in the slopes for the calibration curves is due to the different activity of the electrode surface with low and high concentrations of the analytes. In the lower concentration of the analytes, due to a high number of active sites on the surface of electrode, the slope of the first calibration curve is high. While in the higher concentration of the analytes, due to decreasing active sites on the surface of electrode, the slope of the second segment of the calibration curve decreased, too.

**Fig. 7:**
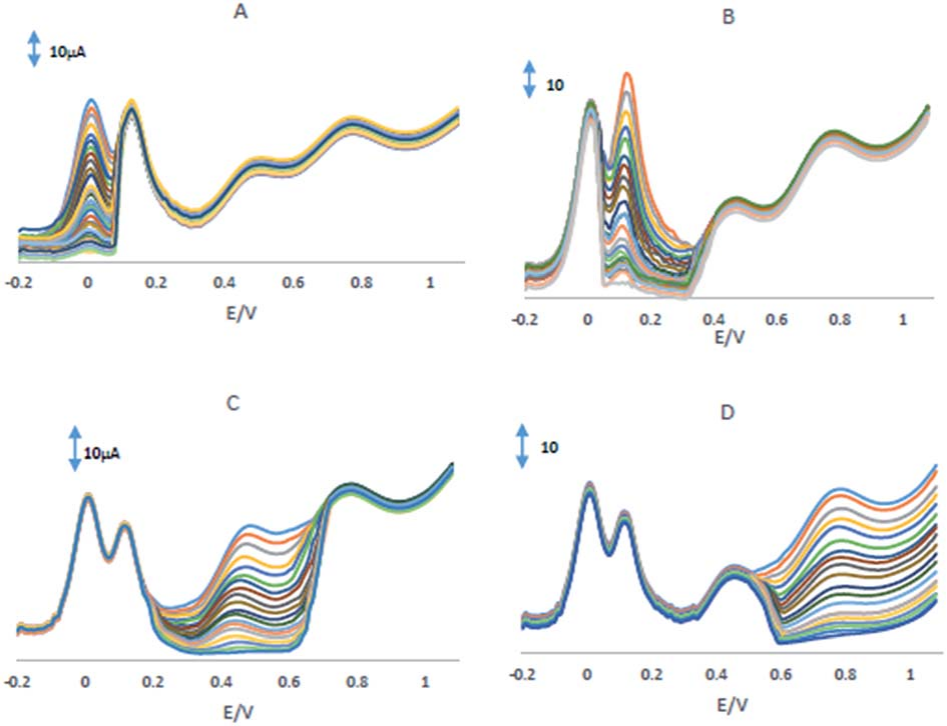
(A) DPVs at the GPE/PQ–BNWs in pH 8.0. PBS, containing (A) 150 μM CC, 500 μM RS and 1 mM NO_2_^−^ and different concentrations of HQ, (B) containing 100 μM HQ, 500 μM RS and 1 mM NO_2_^−^ and different concentrations of CC, (C) containing 100 μM HQ, 150 μM CC and 1 mM NO_2_^−^ and different concentrations of RS, (D) containing 100 μM HQ, 150 μM CC and 500 μM RS and different concentrations of NO_2_^−^.

One of the main problems of electrochemical simultaneous determination of HQ, CC, RS and NO_2_^−^ in presence of each other's is the interferences caused by electrochemically active compounds, which can be oxidized under the same or close potential conditions. In this regard, the mixed solution technique^36^ was employed to study the effect of interfering substances on the simultaneous electrochemical determination of HQ, CC, RS and NO_2_^−^. The electrooxidation processes of HQ, CC, RS and NO_2_^−^ at GPE/PQ–BNWs in the mixture have also been investigated when the concentration of one species changed, whereas those of the other three species are kept constant and the results are shown in Fig. 7. Fig. 7A shows that the peak current of HQ increases by increasing of HQ concentration when the concentrations of CC, RS and NO_2_^−^ are kept constant. Although the charge current was enhanced after HQ was oxidized, the peak currents of CC, RS and NO_2_^−^ did not change (±5%). Similarly and obviously, as shown in Fig. 7B–E, when keeping the concentrations of the other three compounds constant, the oxidation peak currents of CC or RS or NO_2_^−^ were positively proportional to their concentration, while those of the other three compounds did not change. The above results indicate that the proposed sensor is selective for the simultaneous determination of HQ, CC, RS and NO_2_^−^ in present of other compounds exists in real sample.

Also ions of Na^+^, Cl^−^, Zn^2+^, SO_4_^2−^, Ca^2+^, Cu^2+^, K^+^, Mg^2+^ have no interference. The stability of the proposed electrode, GPE/PQ–BNWs, was studied by DPVs peak currents, in repetitive potential scan cycles in different days. The electrode did not show a significant change in the DPV peak currents and potentials of HQ, CC, RS and NO_2_^−^ for more than 5 months; such results could prove the stability of the modified electrode. Also, the stability of the modified electrode was checked for simultaneous determination of HQ, CC, RS and NO_2_^−^. Relative standard deviations (% RSD) for 5 determinations of 20 μM HQ, 30 μM CC, 50 μM RS and 0.1 mM NO_2_^−^ using DPV was less than 0.53%, 0.73%, 1.8% and 3.1%, respectively.

## 3.7. Analytical application

The practical application of the GPE/PQ–BNWs was examined for the simultaneous determination of HQ, CC, RS and NO_2_^−^ in tap water, well water and sea water samples. Based on the results in Table 1, the recoveries for three water samples were 100.4–104.2% for HQ, 98.3–102.7% for CC, 99.4–103.1 for RS and 99.9–103.1% for NO_2_^−^. These amount recoveries are acceptable for determination of HQ, CC, RS and NO_2_^−^. The figures of merit, such as linear range, limit of detection are compared with those from other published works on modified electrodes in Table 2. Based on the data in Table 2, the proposed modified electrode^12,21,37–39^ seems to provide a favorable alternative for the determination of water pollution such as HQ, CC, RC and NO_2_^−^ in real samples with satisfactory results. A simple fabrication procedure, a wide linear range, high stability and good reproducibility for repeated determinations, suggest this electrode being as a good, cheap and an attractive candidate for practical applications. It can be seen the detection limits and the linear ranges for HQ, CC, RC and NO_2_^−^ in our work and other methods in Table 2, that shows detection limits for HQ and CC are lower and linear ranges for the HQ, CC and RS are longer, and for every for samples linear ranges have started from less concentration than former works.

**Table 1:** Determination of HQ, CC, RC and NO_2_^−^ in water samples

Sample		Added (μM)				Found (μM)				Recovery (%)			
		HQ	CC	RS	NO_2_^−^	HQ	CC	RS	NO_2_^−^	HQ	CC	RS	NO_2_^−^
Tap water	1	80	90	150	700	80.1	90.3	150.4	708.1	100.1	100.33	100.26	101.2
	2	150	160	220	1200	148.5	157.2	222.7	1199.2	100.1	98.3	101.2	99.9
Well water	1	80	90	150	700	81.5	90.2	150.1	700.7	101.8	102.7	103.1	100.1
	2	150	160	220	1200	150.7	159.3	220.3	1203.3	100.4	100.8	102.4	100.3
Sea water	1	80	90	150	700	83.4	91.1	151.4	721.1	104.2	101.2	100.9	103.1
	2	150	160	220	1200	153.8	160.2	218.7	1217.1	102.5	100.1	99.4	101.4

**Table 2:** Comparison of the manufactured electrode for HQ, CC, RC and NO_2_^−^ detection with other electrodes

Electrode	HQ		CC		RS		NO_2_^−^		Optimum pH	Ref.
	D.L^a^ (μM)	L.R^b^ (μM)	D.L (μM)	L.R (μM)	D.L (μM)	L.R (μM)	D.L (μM)	L.R (μM)		
CDs/r-GO/GCE	0.17	0.5–1000	0.28	1.0–950	1.0	5.0–600	—	—	7	37
AuNPs/Fe_3_O_4_–APTES–GO/GCE	1.1	3–137	0.8	2–145	—	—	—	—	7.4	38
P-rGO modified electrode	0.08	10–170	0.18	4–50	2.62	10–180	—	—	7	39
MWCNT–SH@Au GR	4.17	54.5–1250.5	1	11–126	7.8	43.5–778.5	23.5	86–7500	7	12
Au/pAMTa–MWNTs/GCE	0.3	7.2–391.2	0.24	3.6–183.6	0.6	8.4–398.4	10	30–419	7	21
GPE/PQ–BNWs	0.12	0.76–362.7	0.2	0.74–321.9	0.82	4.99–970.6	4.5	14.9–2392.6	8	This work

a Detection limit.

b Linear range.

## 4. Conclusions

In this work, for the first time PQ was synthesized from reaction between Q and HQ as bridge and a modified GPE was prepared from PQ–BNWs. This modified GPE/PQ–BNWs was used for simultaneous determination of HQ, CC and RS in presence of NO_2_^−^ as water pollutant. The preparation method is simple, cheap and the proposed modified electrode is very stable. The detection limits for HQ, CC, RS and NO_2_^−^ were 0.12, 0.2, 0.82 and 4.5 μM, respectively. Finally, the GPE/PQ–BNWs successfully was used for simultaneous determination of HQ, CC, RS and NO_2_^−^ in real samples.

## Conflicts of interest

There are no conflicts to declare.

## Supplementary Material
